# Being the Victim of Intimate Partner Violence in Virtual Reality: First- Versus Third-Person Perspective

**DOI:** 10.3389/fpsyg.2020.00820

**Published:** 2020-05-08

**Authors:** Cristina Gonzalez-Liencres, Luis E. Zapata, Guillermo Iruretagoyena, Sofia Seinfeld, Lorena Perez-Mendez, Jorge Arroyo-Palacios, David Borland, Mel Slater, Maria V. Sanchez-Vives

**Affiliations:** ^1^Institut d’Investigacions Biomèdiques August Pi i Sunyer, Barcelona, Spain; ^2^Experimental Virtual Environments for Neuroscience and Technology Laboratory, University of Barcelona, Barcelona, Spain; ^3^Department of Clinical Psychology and Psychobiology, University of Barcelona, Barcelona, Spain; ^4^Institute of Neurosciences, University of Barcelona, Barcelona, Spain; ^5^Department of Cognition, Development and Educational Science, University of Barcelona, Barcelona, Spain; ^6^ICREA, Barcelona, Spain

**Keywords:** virtual reality, embodiment, domestic violence, intimate partner violence, batterer, skin conductance

## Abstract

Immersive virtual reality is widely used for research and clinical purposes. Here we explored the impact of an immersive virtual scene of intimate partner violence experienced from the victim’s perspective (first person), as opposed to witnessing it as an observer (third person). We are ultimately interested in the potential of this approach to rehabilitate batterers and in understanding the mechanisms underlying this process. For this, non-offender men experienced the scene either from the perspective of the victim’s virtual body (a female avatar), which moved synchronously with the participants’ real movements, or from the perspective of an observer, while we recorded their behavior and physiological responses. We also evaluated through questionnaires, interviews and implicit association tests their subjective impressions and potential pre/post changes in implicit gender bias following the experience. We found that in all participants, regardless of perspective, the magnitude of the physiological reactions to virtual threatening stimuli was related to how vulnerable they felt for being a woman, the sensation that they could be assaulted, how useful the scene could be for batterer rehabilitation, and how different it would have been to experience the scenario on TV. Furthermore, we found that their level of identification with the female avatar correlated with the decrease in prejudice against women. Although the first-person perspective (1PP) facilitated taking the scene personally, generated a sensation of fear, helplessness, and vulnerability, and tended to induce greater behavioral and physiological reactions, we show that the potential for batterer rehabilitation originates from presence and identification with the victim, which in turn is more easily, but not exclusively, achieved through 1PP. This study is relevant for the development of advanced virtual reality tools for clinical purposes.

## Introduction

Almost one-third of women worldwide have experienced intimate partner violence (World Health Organization [WHO], 2013). Intimate partner violence is considered by the World Health Organization to be a major public health problem and a violation of women’s human rights. To deal with this large-scale issue, multiple countries have developed intervention programs for batterers and for their victims, the former to reduce the incidence, and the latter to cope with this still-inevitable problem. The intervention programs for offenders, however, have a low success rate and do not have a large effect on recidivism (Gondolf and White, 2001; Shorey et al., 2012b; Radatz and Wright, 2016), the reasons for which include the compulsory nature through which batterers have to complete the programs, non-adherence to the programs, and the heterogeneity of aggressor profiles (Babcock et al., 2005).

Recently, novel technological tools have been elaborated that allow batterers to be virtually in the body of a woman that is being abused. Specifically, Seinfeld et al. (2018) carried out a study in which intimate partner violence offenders embodied, in immersive virtual reality, a female victim that was verbally abused by a male character. The authors observed (1) that offenders have a reduced sensitivity to recognizing female fearful faces, when compared to males without a history of violence, and (2) that the offenders’ emotion recognition skills were improved, especially in their ability to recognize female fearful faces. These findings stress the potential of immersive virtual technology to modulate sociocognitive processes. Moreover, the benefits could be even superior if the virtual scenarios were optimized so that they could be used, in combination with other intervention measures, to reduce the recidivism of offenders.

The technical progress and neuroscience advances concerning body ownership illusions have thus enabled the development of technologies that allow people to be immersed in realistic fictional virtual worlds, and to have the illusion of owning a virtual body (Sanchez-Vives and Slater, 2005; Kilteni et al., 2015; Slater and Sanchez-Vives, 2016). In particular, immersive virtual reality integrates real-time computer graphics, tracking devices to detect body movements, and other appliances to provide sensory inputs that aim to provide a realistic experience in a fictional world. These virtual worlds can include the presence of a virtual body experienced from a first-person perspective (1PP) that incorporates visuomotor synchrony—virtual body movements corresponding to the real movements of the immersed person—and visuotactile synchrony—tactile coherence between the real and the virtual bodies, e.g., through vibrators that are activated when something touches the virtual body. In this sense, immersive virtual worlds provide an opportunity to enter and interact in different scenarios and to experience these as if they were real (Sanchez-Vives and Slater, 2005).

In addition to the article described above in which abusers were put in the virtual body of a female victim of intimate partner violence, immersive virtual reality is already being exploited for research and for clinical purposes. For instance, Stanley Milgram’s obedience experiments (Milgram, 1963, 1974) have been replicated in immersive virtual reality, supporting the potential of virtual worlds to create realistic situations to which people respond as if they were real despite them knowing their fictional origin (Slater et al., 2006; Gonzalez-Franco et al., 2018). These experiments also endorse virtual reality as a tool to overcome the moral and ethical issues arising from studies such as Milgram’s. Another report substantiating this advantage examined sexual arousal and gaze behavior in child molesters that were immersed with adult and child virtual characters showing relevant sexual features (Renaud et al., 2013). The promising therapeutic usage of virtual reality is however not so new: exposure therapy, whereby a threatening agent or context (e.g., a spider) is introduced in a virtual scenario, and which the patient *knows* is not real but *responds to* as if it were real, already revealed supportive results in the mid-1990s and early 2000s (Hodges et al., 1995; Rothbaum et al., 1995; Schultheis and Rizzo, 2001; Garcia-Palacios et al., 2002; Rizzo and Kim, 2005; Gerardi et al., 2008; Powers and Emmelkamp, 2008). The benefits of virtual reality range over a large number of areas (reviewed by Slater and Sanchez-Vives, 2016), and all in all, the increasing use of virtual reality for practical and therapeutic purposes warrants a deeper understanding of the factors that contribute to making the virtual experience seem more real.

One of the factors that modulates the perception of realness of virtual environments is the perspective from which they are experienced: a virtual body witnessed from a 1PP induces physiological reactions and subjective responses to stimuli that are reminiscent of real-life situations, whereas a virtual body observed from a third-person perspective (3PP) does not generate the same physiological changes and subjective sensations, at least not to the same degree (Slater et al., 2010). This has implications on the design of experimental setups since inducing embodiment—leading to the illusion of owning another body—through virtual reality requires a series of devices and considerations that demand greater efforts: embodiment in and out of virtual reality is pronounced when the surrogate body is perceived from 1PP, and is more easily achieved when the design includes visuomotor or visuotactile synchrony (Petkova and Ehrsson, 2008; Perez-Marcos et al., 2009; Slater et al., 2010; Kokkinara et al., 2016). Nonetheless, the findings contrasting 1PP and 3PP described in previous reports need to be validated in experimental designs that are to be used for *real* clinical purposes.

Hence, we compared the impact of an immersive virtual reality scene of intimate partner violence when experienced from the victim’s versus from an observer’s perspective. For this, male participants without a history of violence experienced a virtual reality scene from the perspective of a woman being psychologically abused by a virtual man (1PP condition), or from the perspective of an observer witnessing the same scene (3PP condition), while we recorded their behavior and skin conductance. We also evaluated their subjective impressions after the experience and the change in implicit gender bias. Our findings are relevant for the improvement in the efficacy of virtual reality designs, and contribute to the understanding of the factors that modulate the sensation of realness in virtual worlds, consequently influencing the use of virtual reality for research and rehabilitation.

## Materials and Methods

### Participants

We recruited 32 male participants between 18 and 64 for this study through advertising in the local community. All interviews were carried out by the same psychologist. All participants were explicitly asked whether they had experience of violent situations, and excluded if they answered positively. Half of the participants were assigned to the 1PP condition and the other half to the 3PP condition (Table 1). Participants had no history of violence, no previous experience or knowledge of computer programming, they were not regular videogame users and they had not previously taken part in any study involving virtual reality. Other exclusion criteria included whether the subject had ignored the virtual man and/or the scene. Due to this, we excluded three subjects in the 1PP condition and two in the 3PP condition. This led to a final sample size of *N* = 13 for the 1PP condition and *N* = 14 for the 3PP condition. All subjects gave written informed consent and were paid 10€ for their participation. This study was approved by the Ethics Committee of the Hospital Clínic de Barcelona and was carried out according to the Declaration of Helsinki (World Medical Association, 2013).

**TABLE 1:** Sociodemographic information of the participants assigned to the first-person and to the third-person conditions.

	First person	Third person	Statistics
*N*	13	14	
Age (SD)	31.8 (9.1)	31.36 (7.2)	*t* = 1.56, *p* = 0.88
Education*	*U* = 61.5, *p* = 0.13		
No studies	0	0	
Primary	1 (8%)	0	
Secondary	1 (8%)	0	
Vocational training	4 (31%)	1 (7%)	
University	3 (23%)	8 (57%)	
Post-university	3 (23%)	3 (21%)	
Ph.D.	1 (8%)	2 (14%)	
Country of origin	Fisher’s exact test: *p* = 0.42		
(Spanish/Not Spanish)	10/3	8/6	
Years in Spain (SD)	7.5 (11.7)	4.67 (5.3)	*U* = 7.5, *p* = 0.70
Working (yes/no)	7/6	6/8	χ^2^ = 0.326, *p* = 0.57

*^∗^Percentages have been rounded up.*

### Study Design

**FIGURE 1:**
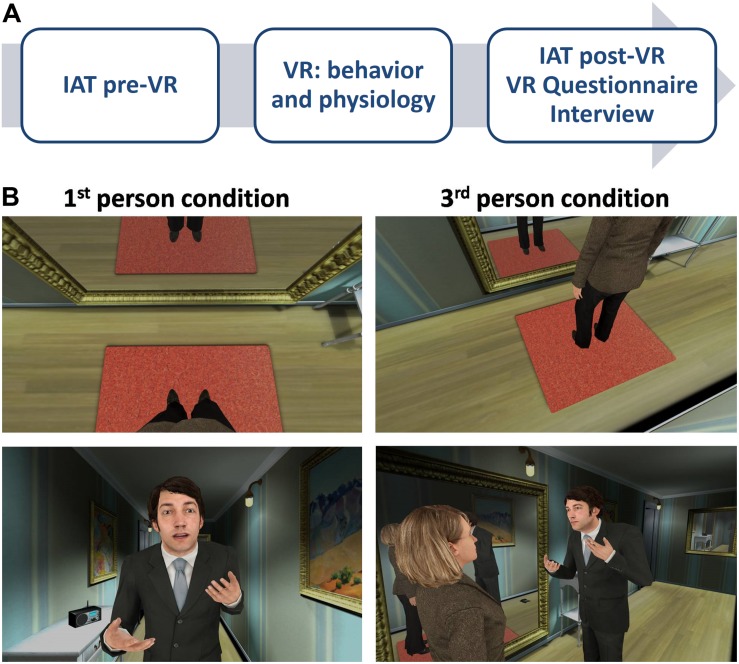
Study design. **(A)** Experimental procedure. **(B)** Extracts of the virtual reality scene. Left, first-person perspective, in which the participant could see the virtual body of a woman replacing his own body when he looked down or in the mirror (top) and interacted face-to-face with the virtual male abuser (bottom). Right, third-person perspective, in which the participant could see a virtual woman (top) interacting with the virtual male abuser (bottom) as an observer. IAT, implicit association test; VR, virtual reality.

To determine whether a virtual reality scene of intimate partner violence experienced from the victim’s perspective (Seinfeld et al., 2018), as opposed to witnessing it as an observer, could be better used to rehabilitate abusers, we studied 32 participants who underwent a similar procedure except that they were assigned to either the 1PP or the 3PP condition (Figure 1A). All participants first put on a suit for body tracking, and then completed the Implicit Association Test (IAT, pre-VR, pre-virtual reality) to explore their baseline implicit gender bias. The sensors for the physiological recordings and the head-mounted display (HMD) were subsequently set up. Participants were then immersed in a virtual hallway of a house, where they experienced an abuse scene of a man toward a woman, either from the body of the female victim (1PP condition) or as an observer (3PP condition). In the 1PP condition, subjects embodied a virtual woman that moved according to the participants’ movements. They could also see their virtual female body reflected in a mirror in front of them moving in synchrony with their own body (Figure 1B, left). In the 3PP condition, subjects observed the same scene but from the perspective of an observer placed next to the female victim, from an eye level point of view and without a virtual body (Figure 1B, right). We analyzed the behavior of the participants and we recorded their electrocardiogram (ECG) and galvanic skin response (GSR) during baseline and throughout the virtual abuse scene. The abuse scene consisted in a virtual man psychologically abusing the virtual woman as in Seinfeld et al. (2018). At some point, the virtual man hit a telephone, after which he approached the victim in a threatening manner, ending up very close to her, invading her personal space, as he went on speaking, appearing more intimidating as time went on. In the 1PP condition, the experimenter could make the virtual man say some predefined sentences (see section “Virtual reality scenario” below) to enrich the virtual man-woman interaction. The abuse scene lasted 3 min. Once finished and after removing the HMD and the other devices, participants completed the IAT (post-VR) to detect changes in implicit gender bias after having lived through the virtual scene, and completed a virtual reality experience questionnaire to assess the participants’ subjective impressions of their experience. Lastly, we interviewed the subjects to gain further insights into their experience in virtual reality (Figure 1A).

### Virtual Reality Setup

The virtual scenario was presented in an NVIS nVisor SX111 HMD featuring two screens with a resolution of 1280 × 1024 pixels and 76°H × 64°V field-of-view (FOV) per eye, updated at 60 Hz. Both screens were positioned with an angle facing outward, with 50° horizontal overlap (66%), achieving a combined FOV of 102°H × 64°V. Head tracking was attained with an Intersense IS-900 device with six degrees of freedom.

A tight OptiTrack suit with reflecting markers was used to track whole body movements. The tracking system worked via infrared signals and consisted of a 12-camera OptiTrack system (NaturalPoint) that can track an approximate volume of 2.5 × 2.5 × 3 m high. The Arena OptiTrack software to capture movements was employed to reconstruct the dynamic skeletal configuration of the participants from the data in the camera at 100 Hz with millimeter accuracy. This skeletal information was transferred onto the virtual reality through the NatNet streaming protocol, and was assigned to the virtual avatar so that the posture and movements coincided with those of the participants. The software used was XVR for the overall virtual reality system (Tecchia et al., 2010), and HALCA for the animation of characters (Gillies and Spanlang, 2010).

### Virtual Reality Scenario

Once the HMD and the sensors were set up, participants were asked not to walk around the room, although they were allowed to move their arms, hands and legs, as well as move within one square meter. Real-time head and body tracking was used in both conditions. In the 1PP condition, participants had their bodies replaced with that of a virtual woman, which was co-located with their real body and which moved synchronously with their real movements thanks to visuomotor synchrony (Figure 1B, left). In the 3PP condition, participants saw the virtual woman from a third-person perspective, without a body (Figure 1B, right).

At this point, participants were immersed in a virtual scenario (Seinfeld et al., 2018). They stood at one end of a virtual hallway that was about 2 m wide by 9 m long. In the hallway, looking toward the other end, there was a full length mirror on the left wall, a small table to the right of the mirror with a radio and a telephone on it, an open door at the end of the left wall, a smaller hanging mirror on the front wall, a few pictures and a 1 × 1 m carpet on which the virtual woman stood, right in front of the full length mirror (Figure 1B, bottom). This carpet was used as a reference point so that the participants could identify the area within which they could freely move. We made sure that the image in the HMD was properly focused and we then asked participants to describe their surroundings to confirm that they paid attention to the most important details: the large mirror to their left, and the radio and telephone on the table. A 2-min baseline period followed in which subjects stayed still, relaxed and looking toward the closer end of the hallway, where there were no objects, while their baseline physiological measures were recorded.

The baseline recordings were followed by 2.5 min of stretching exercises. Participants in the 1PP condition had to follow a set of instructions coming from the radio on the table (so were they told) in order to develop a greater sense of ownership of the virtual woman’s body, whereas participants in the third-person condition merely observed the virtual woman doing the exercises while they also simultaneously heard the radio instructions. Emphasizing the visuomotor synchrony through the stretching exercises—consisting in head, arm and leg movements—allowed the subjects in the 1PP condition to become familiarized with and be embodied in, the virtual female body. On the other hand, those in the 3PP condition were asked to face the open door at the end of the hallway; they were told that the image would vanish and that, when it showed up again, they would see a woman doing exercises in front of a mirror, but they did not have to copy those exercises. The perspective in the 3PP condition was arranged so that participants would see the abuse scene from the right side of the virtual woman, including her in the view.

The virtual male abuser then entered the room and started to verbally abuse the virtual woman following a predefined script (Seinfeld et al., 2018) designed for psychological abuse, constantly looking at her in the eyes. At some point, the virtual man hit the telephone sitting on the table in the hallway and threw it to the floor in the woman’s direction, thus affording an act of physical violence. In the 1PP condition, the experimenter had slight control over the virtual man throughout the scene, since he could make the virtual man say “Look at me!” when the participant looked away, or “Shut up!” and “I told you to shut up!” when the participant spoke, which facilitated the virtual man-woman interaction.

### Video Analysis

We videotaped the participants while they were immersed in the virtual reality scenario to analyze their reactions to the male avatar: we counted the number of times that participants directly spoke to the male avatar—without being asked to do so—and their verbal and non-verbal behavior in the following situations: (1) when the male avatar questioned the woman’s appearance by saying “But have you seen yourself? Just look at you!”; (2) when the male avatar threw the phone to the floor in the woman’s direction; and (3) when the male avatar approached the woman after throwing the phone, in a threatening manner, invading her personal space. Participants were not provided with any information or rules regarding speaking or not speaking to the male avatar, how to move, etc., except for being advised to stay within one squared meter in order not to yank wires or lose their HMD.

### Physiological Measures

We recorded physiological parameters—skin conductance—at baseline and while participants were immersed in the virtual reality scenario as an objective measure of stress/arousal. Physiological data were obtained with a wireless g.MOBIlab device (g.tec). The sensors included a g.GSR sensor, with electrodes placed on the index and middle fingers to register the galvanic skin response (GSR). We compared the GSR between baseline and the reactions to two selected events between the two groups: when the male avatar threw the phone, and when he approached the woman in a threatening manner right after throwing the phone. For this, we set triggers on the recordings at the onset of each of these two events. Baseline recordings (2 min) were obtained before starting the experiment and were used to standardize the GSR. The GSR is expected to increase after exposure to threatening stimuli as it reflects arousal (Cacioppo et al., 2017) and has in fact been found to correlate with the strength of illusory body ownership (Ehrsson et al., 2007).

From the physiological signals, we took windows of 35 s zero-centered at the “man throws phone” event which is the first trigger (see Figure 3A). We standardized the data by subtracting the mean of the signal in the 5 s before the first trigger and by dividing it by the standard deviation of the baseline. Responses were quantified by obtaining the area under the curve (AUC) following each of the triggers. As we set values to zero before the first event, responses of both events are referenced to this moment, and the second event response is influenced by the response to the first. The AUC for the GSR was computed from 2 to 9 s after each trigger.

### Gender Implicit Association Test (IAT)

We administered a gender version of the IAT (Greenwald et al., 1998) to identify the level of the participants’ implicit (automatic) gender bias before (pre-VR) and after (post-VR) the virtual abuse scene experienced from the victim’s or an observer’s perspective. In this gender IAT, participants had to quickly categorize certain attributes (e.g., generosity) with pairs of words (e.g., woman – good vs. man – bad). The differences in accuracy and reaction times were used to compute the implicit gender bias, since people tend to categorize words into the same group more easily and faster when they belong to closely related categories. Positive IAT scores correspond to a preference for pairing positive words with man, and negative IAT scores correspond to a preference for pairing positive words with woman.

The specific attributes to be paired with man vs. woman, good vs. bad, or with the pairs of words man-good vs. woman-bad, and woman-good vs. man-bad, were the following: niece, sister, girl, mother, feminine, wife, woman, aunt, daughter, nephew, brother, boy, grandfather, father, masculine, husband, man, uncle, son, generosity, relaxed, happy, glory, pleasant, peace, good, wonderful, smile, joyful, agony, disgusting, mistake, horrible, unpleasant, cry, frightening, pain, evil, and terrible. Similar versions of the IAT have been used by Eckhardt et al. (2013) and Jin (2016).

### Virtual Reality Experience Questionnaire (VR Questionnaire)

After the IAT (post-VR) and before the final interview, we administered an in-house questionnaire to assess the subjective experience of the participants during the virtual reality scene (see Figure 5, top). The questions were to be answered on a 7-point Likert scale in which 1 meant “not at all” and 7 meant “very much.” The VR questionnaire comprised questions related to the sense of ownership (*identification*, *in body*), the virtual man-woman interaction (*personal*), the fact of being a woman instead of a man (*man mirror*, *vulnerable woman*), the danger of the situation (*assaulted*), the wish to respond to the virtual man (*wish assault*), the degree of difference between virtual reality and television (*TV*), the potential of gaining a new perspective (*diff view*) and the potential to use virtual reality to rehabilitate batterers (*use VR*). We grouped and analyzed related questions in three separate groups: woman’s body (*identification*, *in body*), take personally (*assaulted*, *personal*) and vulnerable (*man mirror*, *vulnerable woman*); and we analyzed the other 4 unrelated questions independently (*wish assault*, *TV*, *diff view*, *use VR*) (see section *Statistical analysis* below).

### Interview

We interviewed the participants at the very end of the experimental procedure to evaluate qualitatively their feelings after the virtual reality scenario. The interview lasted between 5 and 15 min. All questions were open-ended and they attempted to amplify, qualify or delve into some of the responses or comments that had been mentioned during the virtual reality questionnaire.

Interviews were videotaped, transcribed and analyzed. The qualitative analysis was carried out following MAXQDA software’s methodology using a code system based on a tree structure (MAXQDA, VERBI GmbH, Berlin, Germany). In short, the transcribed interviews were read, categorized (classification of concepts within topics), subcategorized (separation into segments mentioning features, properties or dimensions), and codified (assignation of labels/codes for each segment that was part of a category) by the experimenter; we provide the outcome of the interview as additional descriptive information to complement the quantitative findings.

### Statistical Analyses

As a first step, we examined whether the data were normally distributed with the Shapiro–Wilk test, which was done separately for each group of participants. We removed far-out outliers that were more than 3^∗^IQR from the median (only one participant in the first-person condition in the skin conductance response, and another one in the third person condition in the IAT, both of which were included for the other analyses). We used the Chi-squared test to contrast categorical demographic variables (working status, behavioral responses of the videos), and we used the Fisher’s exact test when the assumptions for the Chi-squared test were violated (country of origin, behavioral responses of the videos). For non-normally distributed continuous (years in Spain) and ordinal variables (education), we used the Mann–Whitney *U* test, and for normally distributed continuous variables (age) we used independent *t*-tests. Repeated measures ANOVAs were employed for the questionnaire, which we divided into three groups of two related questions (woman’s body: *identification* + *in body*, take personally: *assaulted* + *personal*, vulnerable: *man mirror* + *vulnerable woman*), each analyzed with a separate ANOVA, with the variables *question* (two questions in each group of questions) as the within-subjects variable and *condition* (first vs. third person) as the between-subjects variable (Norman, 2010; Sullivan and Artino, 2013). *Post hoc* tests (corrected for multiple comparisons; Benjamini and Hochberg, 1995) and comparison of the other four questions in the questionnaire (*wish assault*, *TV*, *diff view*, *use VR*) were carried out with the Mann–Whitney *U* test. Another repeated measures ANOVA with *time* (pre- vs. post-virtual reality) and *condition* (first vs. third person) was run to compare IAT scores before and after the virtual abuse scene, and another one with *event* (man throws phone vs. man gets closer) and *condition* (first vs. third person) was used to contrast the physiological reactions across events and between groups. Non-parametric Spearman correlation analyses were run to evaluate the relationship between the physiological reactions to the two threatening events, the subjective impressions of the virtual experience (virtual reality questionnaire), and the change in gender bias (IAT post-VR minus IAT pre-VR). Statistical significance was set at *p* < 0.05. All analyses were carried out in SPSS 20 (IBM Corporation, Chicago, IL, United States).

## Results

Based on the video analysis we excluded five participants (see *Participants* in section “Materials and Methods”). Participants in the first-person and in the third-person conditions did not differ in terms of age, education, country of origin and working status at the time of the study (Table 1).

### Behavioral Analysis

**FIGURE 2:**
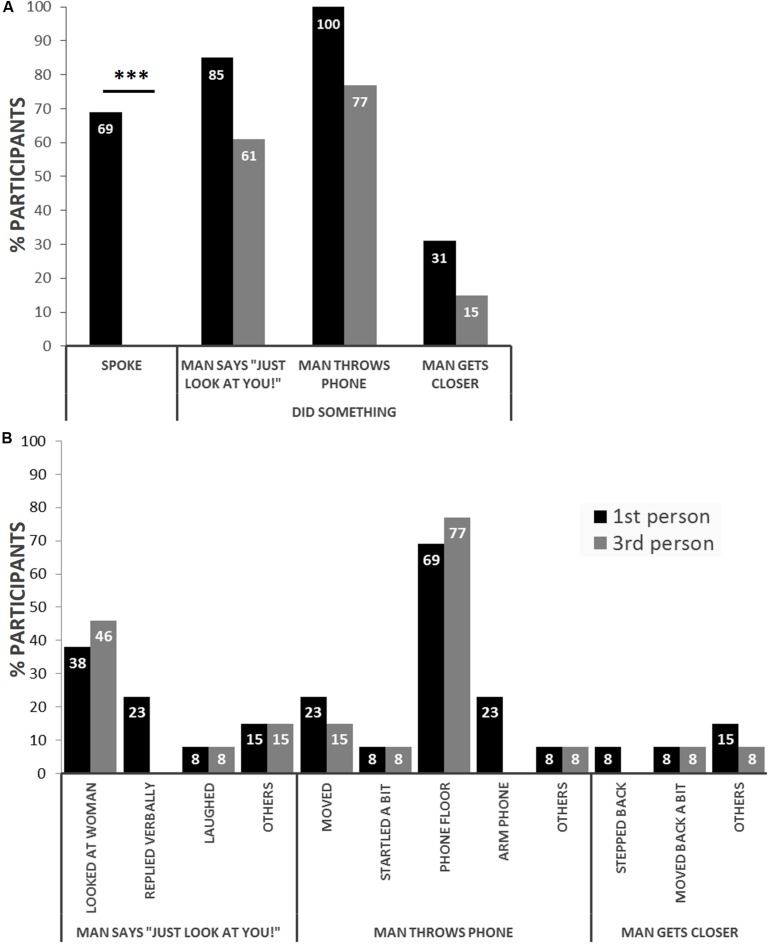
Experiencing a virtual abuse scene from the perspective of a female victim induces greater behavioral reactions than does experiencing it as an observer. **(A)** Behavioral analysis showing that participants in the victim’s perspective (first person) spontaneously spoke more to the virtual man (*spoke*) and tended to react more in general to the 3 events of interest: when the virtual man questioned the woman’s appearance (*man says “Just look at you!”*), when the man threw the phone in the woman’s direction (*man throws phone*) and when the man approached the woman in a threatening manner (*man gets closer*). **(B)** Behavioral analysis breakdown showing the types of behaviors in response to the three events of interest. For clarification: *looked at woman* = looked down to where their body would be or their reflection in the mirror (first person) or moved head to purposefully look at woman (third person); *moved* = moved the body; *phone floor* = followed the trajectory of the phone all the way to the floor; *arm phone* = followed the trajectory of the man’s arm until he hit the phone but not the phone to the floor; *others* include biting lips, smiling, putting hands on waist, muttering (man says “Just look at you!”); slightly moving head, laughing (man throws phone); moving hands, trying to touch virtual man (man gets closer). ***p < 0.001.

Participants who experienced the domestic violence scene in immersive virtual reality from the 1PP of a female victim reacted to a greater extent than those who experienced the same scene from a 3PP, as observers (Figure 2). The only significant difference, however, was in the number of times participants spontaneously spoke to the virtual man without being asked to do so, which happened in 9 out of 13 participants in the first-person condition, and 0 out of 13 (one video missing) in the third-person condition (*p* < 0.001; Figure 2A). The remaining parameters showed trends, albeit not significant, in the same direction: more participants in the first-person condition reacted to the 3 events of interest *in general* (Figure 2A), and relatively similar reactions to the 3 events of interest were observed in both conditions *in particular*, with participants in the 1PP condition tending to react more “personally,” e.g., by replying verbally to the virtual man when he questioned her appearance (*virtual man says “Just look at you!”*) and by stepping back when the virtual man approached her in a threatening manner (*man gets closer*) (Figure 2B).

### Physiological Measures

**FIGURE 3:**
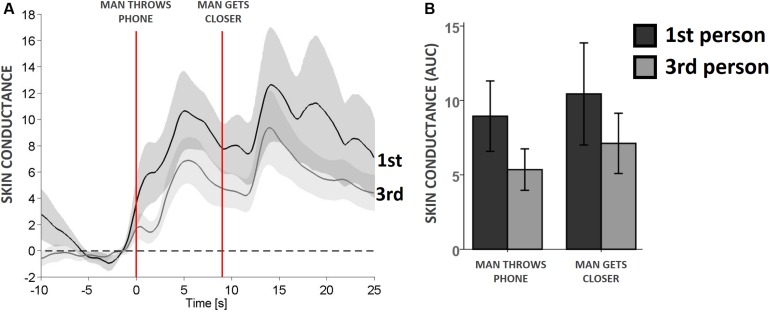
Experiencing a virtual abuse scene from the perspective of a female victim induces slightly stronger physiological reactions to threatening stimuli than does experiencing it as an observer. **(A)** Skin conductance response of participants assigned to the victim’s (first, dark gray) and an observer’s (third, light gray) perspectives around two selected virtual threatening events: when the man throws the phone in the woman’s direction (onset at leftmost red vertical line), and when the man gets closer to the woman in a threatening manner (onset at rightmost red vertical line). Line, average; shade, standard error. **(B)** Quantification of the skin conductance response at the two events. ANOVA did not reveal significant differences. AUC, area under the curve (see section Materials and Methods: Physiological Measures for details).

There was a clear physiological reaction to the two selected threatening events: when the virtual man threw the phone and when he approached the virtual woman shortly after throwing it. The physiological reaction was visible as an evident increase in the GSR (Figure 3A); despite the clear reaction, the two groups did not differ significantly from one another even though, similarly to most of the parameters we evaluated, there was a trend pointing to a greater skin response in the participants who experienced the abuse scene from a first-person perspective (Figure 3B).

### Implicit Association Test (IAT)

**FIGURE 4:**
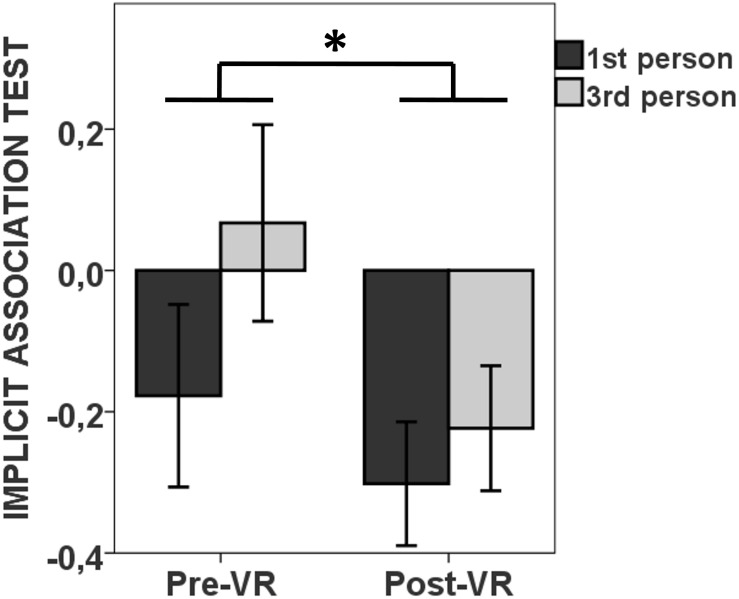
Witnessing a scene of male abuse toward a woman in virtual reality reduces the prejudice against women. Experiencing the scene from the perspective of the victim (first person) or as a mere observer (third person) resulted in a decrease in IAT scores, without a group effect or interaction, implying a reduction in prejudice against women. Pre-VR and Post-VR, before and after the virtual abuse scene, respectively. *p < 0.05.

In terms of implicit gender bias toward assigning certain positive or negative attributes more often to either men or women, we did not find differences between groups neither at baseline nor after the virtual scene, although witnessing the virtual domestic violence scene reduced the prejudice against women in both groups, independently of perspective [main effect of *time*: *F*(1,24) = 5.58, η^2^ = 0.189, *p* = 0.027] (Figure 4).

### Virtual Reality Questionnaire

**FIGURE 5:**
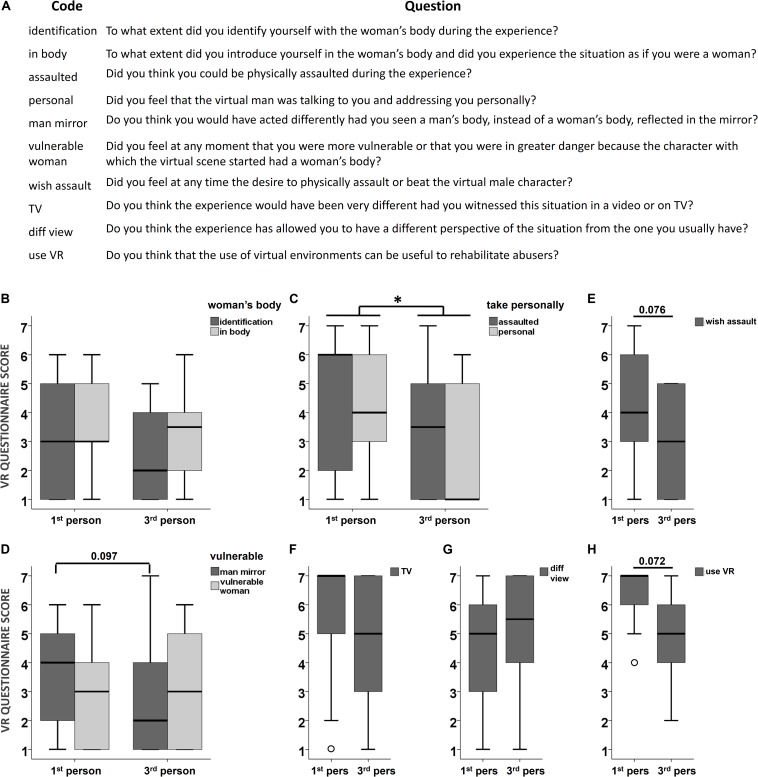
Experiencing a virtual scene of intimate partner violence from the victim’s perspective facilitates taking the experience personally. **(A)** Questions in the virtual reality questionnaire and their assigned codes depicted in **(B–H)**. All questions were rated on a Likert scale ranging from 1 (not at all) to 7 (very much). **(B–H)** Results of the virtual reality questionnaire administered after the virtual abuse scene to participants who experienced the scene from the victim’s perspective (first person) or as mere observers (third person). Boxplots represent median and 95% CI. ^∗^*p* < 0.05.

We found differences in how participants rated their experience in virtual reality depending on the perspective from which they witnessed the domestic violence scene, with the 1PP perspective inducing higher (or a tendency to higher) ratings in almost all questions (Figure 5). The ANOVAs revealed that participants in the 1PP condition lived the scene more personally [i.e., *take personally*: *personal* and *assaulted;* main effect of *condition*: *F*(1,25) = 6.26, η^2^ = 0.200, *p* = 0.019] (Figure 5B); that they would have acted differently to a higher degree than participants in the 3PP condition had they been a man in the virtual scene [*vulnerable*: *question* × *condition* interaction: *F*(1,25) = 4.24, η^2^ = 0.145, *p* = 0.050; *post hoc man mirror*: Mann–Whitney *U* = 57.50, *p* = 0.097] (Figure 5C); and two trends also suggesting that participants in the 1PP condition had a greater wish to assault the male abuser (*wish assault*; Mann–Whitney *U* = 54.50, *p* = 0.076) (Figure 5E) and that they considered virtual reality to be more useful for batterer rehabilitation (*use VR*; Mann–Whitney *U* = 49.0, *p* = 0.072) (Figure 5H). We did not find significant effects or interactions in the other questions (all *p* > 0.1). Overall, these findings suggest that participants who experienced the victim’s perspective lived the scene more personally than those who merely witnessed it as observers.

### Relationship Between Physiological Responses, Subjective Experience and Gender Bias

Interestingly, the magnitude of the physiological reactions to the two selected threatening events correlated with several of the virtual reality questionnaire scores (Table 2), and this occurred when all participants were pooled together but not when they were separated by condition, probably because the sample size in each group separately was too small to reach significance. In particular, stronger skin conductance responses to the two threatening events were coupled with higher ratings on how much participants thought they would have acted differently had they been a man (*man mirror*), how much they thought they were more vulnerable for being a woman (*vulnerable woman*), how much they thought they would be physically assaulted (*assaulted*, only *man gets closer*), how much they thought the experience would have been different had they seen it on TV (*TV*, only *man throws phone*), and how much they thought this virtual paradigm could be useful to rehabilitate batterers (*use VR*).

**TABLE 2:** Relationship (Spearman correlation) between skin conductance responses to two threatening events, scores on the virtual reality questionnaire (see Figure 5 for each item) and change of implicit gender bias (a negative value of IAT Post-Pre indicates a decrease in prejudice against women after the virtual scene).

All participants	Skin Conductance Phone	Skin Conductance Man Closer	All participants	IAT Post-Pre
Man mirror	0.438^∗^	0.481^∗^	In body	−0.389^∗^
	0.025	0.013		0.045
Vulnerable woman	0.595^∗∗^	0.597^∗∗^		
	0.001	0.001		
Assaulted		0.442^∗^		
		0.024		
TV	0.484^∗^			
	0.012			
Use VR	0.470^∗^	0.515^∗∗^		
	0.018	0.008		

*Top value, Spearman coefficient ρ; bottom value, significance p. ^∗^p < 0.05, ^∗∗^p < 0.01. IAT, implicit association test; VR, virtual reality.*

In addition, the degree to which participants experienced the virtual scene as if they were a woman (*in body* question) was coupled to the amount of change in implicit gender bias: stronger feelings that they were the woman were linked to a larger reduction in the IAT, implying a larger decrease in prejudice against women (Table 2). This correlation held for all participants pooled together, and the correlation was even higher in the first-person condition when the analysis was performed separately for each group (Spearman ρ = -0.61, *p* = 0.028).

### Interview After the Virtual Reality Experience

Based on a qualitative analysis of the interview after virtual reality, we found that experiencing the virtual domestic violence scene from a 1PP and from a 3PP resulted in divergent perceptions and opinions (Table 3). Overall, participants who experienced the scene from the perspective of the female victim tended to consider the experience more real, more threatening, with a high potential to empathize with a female victim of domestic violence, with more intense feelings of fear, helplessness, alertness and vulnerability, and mentioned its potential application as a rehabilitation approach for domestic violence assaulters. On the other hand, participants who experienced the scene as observers lived through the virtual environment in a more detached manner, had a less threatened impression although they did feel uncertainty, anxiety and repulsion, considered the experience useful to develop empathy skills and mentioned the option of being immersed in the woman’s body as a possibility to increase its potential.

**TABLE 3:** Summary of the qualitative analysis of the interview carried out after the virtual reality scene in participants who experienced the virtual abuse scene from the perspective of a female victim (first person) and as an observer (third person).

First person	Third person
Participants were integrated in an environment that facilitates empathy and a change in perspective. They consider that virtual reality can be useful for personal improvement and for skill development.	Participants experienced the virtual environment with some detachment, although it allowed them to be immersed in the situation but as observers.
Participants felt in a state of alert (more than threatened) and uncomfortable for not keeping a distance (with aggressor).	They did not have a threatening sensation, but they did feel uncertainty and some fear to the unknown.
They think that the experience can turn out to be useful to rehabilitate abusers because it facilitates a change in empathy (change in perspective) and it invites to reflect and reconsider, to discern.	Participants think that the experience can be useful to develop interpersonal skills and to increase empathy.
They consider virtual reality as: relatable, first-person experience, immersed (enveloped), introduced (integrated), characters less real than on TV.	They consider virtual reality as: surrounding, to be inside of.
Emotions: helplessness and fear.	Emotions: being alert and repulsion.
Received input: intense experience and empathy.	Received input: reminder of the domestic violence problem, rejection of male chauvinism and greater sensitization toward the victim.
Feelings: rage, powerlessness, insecurity, vulnerability, fear, nervousness and tension.	Feelings: a certain state of generalized anxiety.
Final comment: sensation of reality, short duration and new way toward the future.	Final comment: it could be more interesting if you were immersed in the woman’s body.

## Discussion

In this study we found that an immersive virtual reality scene of intimate partner violence experienced from the victim’s perspective, and to a lesser degree witnessed as an observer, could be a useful tool to be included in intervention programs to rehabilitate abusers. The degree to which subjects feel part of the scene seems to contribute more to the potential of this virtual reality paradigm rather than only the perspective from which they experience it. This is evident from the correlation analyses revealing, in all participants, a linear relationship between the magnitude of the physiological reactions to virtual threatening stimuli and their subjective impressions of the experience assessed through a questionnaire. Furthermore, a higher identification with the virtual woman was coupled to a greater decrease in prejudice against women after the scene. Experiencing the virtual scene from 1PP facilitated the identification with the woman and induced a sensation of fear, helplessness, alertness and vulnerability, which is likely the reason for which participants in the victim’s perspective tended to react more to the scene, in terms of behavior and physiology, and scored (or tended to score) higher in the virtual reality questionnaire. In other words, we show here that the great potential of this virtual abuse scene for the rehabilitation of intimate partner batterers originates from being immersed in the virtual environment, which in turn is more easily, but not exclusively, achieved through 1PP. It also has a potential use to target public awareness and education to decrease victim-blaming attitudes in society (Gracia, 2014). Research on these issues is clearly insufficient since important research questions remain open. This study is relevant for the improvement in the efficacy of virtual reality designs, and contributes to the understanding of the factors that modulate the sensation of realness in virtual worlds, consequently benefiting the development of advanced virtual reality tools to be used for clinical purposes.

This is not the first study to show that experiencing immersive virtual reality from a 1PP is different from experiencing it from a 3PP; it is however the first to look at this in a paradigm to be potentially used for clinical purposes, in particular for the rehabilitation of intimate partner batterers. In previous work, Slater et al. (2010) showed that men that witnessed a virtual scene from the 1PP perspective of a girl could have body ownership with respect to the girl’s virtual body, perceived the situation as if they were the girl, and showed stronger physiological reactions to threatening virtual stimuli than did participants who experienced the same scene from 3PP. In agreement with their findings, we found here that in the 1PP condition the physiological response to stressful events tended to be larger than in the 3PP condition. In the 1PP condition participants significantly took the scene more personally, as suggested by stronger feelings that they could be physically assaulted (*assaulted*) and that the virtual male abuser was talking to and addressing them personally (*personal*), that their reaction would have been different had they been a man instead of a woman (*man mirror*), that they wished to assault the virtual male abuser (*wish assault*) and that this virtual scene would be useful for the rehabilitation of intimate partner assaulters (the last three instances being non-significant trends, however; see Figure 5), together with the more evident behavioral and physiological reactions (see Figures 2, 3).

Nonetheless, the major differences between groups seem to be due to a linear pattern and not so much to a binary factor. In other words, it seems that it was not exclusively the different perspectives that induced the group differences, but a linear relationship between the levels of presence and identification with the virtual woman that corresponded to equivalent levels of physiological reactions and the decrease in prejudice against women. The scores in the virtual reality questionnaire were high in both groups, albeit slightly lower in the third-person condition. This implies that, because participants experienced the scene from the perspective of the victim or of an observer (either 1PP or 3PP), but not both, their presence (place location and plausibility) in a virtual reality environment was enough to induce a sensation of realness (Slater, 2009). This is supported by our findings that both groups showed (1) physiological reactions to threatening virtual events, that is, they reacted physiologically as if the virtual situation were real (Petkova and Ehrsson, 2008; Kilteni et al., 2012), (2) behavioral responses throughout the virtual abuse scene, both of which (physiology and behavior) were less pronounced but still existent in the third-person condition, and (3) a decrease in gender bias, i.e., a reduction in prejudice against women, in all participants regardless of the perspective, after witnessing the scene of intimate partner violence. The qualitative analysis of the interview further illustrates how participants, especially those who underwent the victim’s perspective, reacted to the virtual scene as if it were real, as they described a sensation of fear, helplessness, alertness and vulnerability, among others (see Table 3). Furthermore, the fact that larger skin conductance responses to threatening virtual stimuli were coupled to a higher sense of vulnerability, fear of being assaulted and a stronger belief of the potential use of virtual reality for rehabilitation, and that a greater feeling of being a woman was related to a larger decrease in prejudice against women, in all participants irrespective of perspective, highly suggests that the main factors contributing to the potential success of this virtual paradigm are not necessarily the perspective through which it is experienced, but the degree to which the subjects are immersed in the virtual scene and identify themselves with the virtual female body; this is however more easily, but not exclusively, achieved through a first-person perspective.

Another one of the mechanisms that can contribute to the usefulness of our immersive paradigm for the rehabilitation of intimate partner batterers is that we detected a decrease in gender bias after witnessing the virtual scene. These findings, together with a recent study that used the same paradigm (in first-person mode) to show that intimate partner abusers improved their recognition of female fearful faces and decreased their bias toward wrongly attributing a happy emotion to female fearful faces (Seinfeld et al., 2018), emphasizes the potential of virtual reality tools, and in particular of our paradigm, to be tested in, and eventually applied to, clinical or forensic populations. The potential of virtual reality paradigms to induce modifications in perceptions, attitudes and even beliefs has been demonstrated in multiple instances and is therefore not surprising that we obtained comparable results. For example, the sensation of owning a body that belongs to an outgroup, be it race, age or gender, achieved by co-location of the real and a virtual body in immersive virtual reality, reduces the implicit biases against this outgroup (Slater et al., 2010; Banakou et al., 2013, 2016; Peck et al., 2013). Maister et al. (2015) proposed that this change in implicit bias takes place in two steps: first, through a physical, bodily relatedness with the new form that makes the new *self* similar to the outgroup; and second, through the extension and generalization of this relatedness and similarity to positive *self*-like associations to the outgroup. This reduction in implicit bias against outgroups in which participants are embodied has been repeatedly demonstrated (Banakou et al., 2013, 2016; Peck et al., 2013). A neural network model has been proposed that also well-simulates these findings (Bedder et al., 2019). Altogether, these and our study corroborate that immersive virtual reality can change the implicit associations, attitudes, perceptions and even behavior of participants, and thus highlight its potential as a powerful tool for psychology research and for psychotherapy, entailing that it should be well comprehended to increase its potential.

The use of immersive virtual reality designs for clinical purposes is not new (e.g., Hodges et al., 1995 and many others) with evidence showing that results are at least as good as face-to-face interventions (Adamovich et al., 2009). Virtual paradigms have already been tested, proven to be favorable and even become a commonly used approach to treat psychological issues such as phobias (Powers and Emmelkamp, 2008). Other studies have focused on its potential to treat or ameliorate the symptoms of Parkinson’s disease (Mirelman et al., 2013), to help in the recovery of motor function after brain injury (Levin et al., 2015) and other types of neurorehabilitation (Perez-Marcos et al., 2012), and on the assessment of patients with neurological conditions (Llobera et al., 2013). Here we provide evidence for the potential of our virtual reality design to complement rehabilitation programs for intimate partner batterers, given that it provides subjects with a novel realistic perspective with which to empathize and it decreases prejudice against women. Moreover, the questionnaire scores and the interview after the virtual experience substantiate the high potential of this tool (see Figure 5D and Table 3). In fact, as highlighted above, Seinfeld et al. (2018) have already reported some of the benefits of this paradigm in a study with intimate partner batterers.

In most cases, rehabilitation programs of intimate partner offenders use multiple components identified in different theoretical models. Some of these programs include the Duluth Model, cognitive-behavioral therapy or new mindfulness-based intervention called Achieving Change Through Values-Based Behavior which was designed for court-mandated men or women who engage in intimate partner violence (Snead et al., 2018). In all cases, the contribution of virtual reality as a therapist’s tool for deepening the induction of emotional and cognitive changes and incorporating fair and healthy attitudes and behaviors within relationships is feasible. The combination of virtual reality with other tools can thus be integrated in a rehabilitation program for men who are in or out of prison, in the same way that current rehabilitation programs also target men in and out of prison.

In spite of these promising findings there are some caveats. First of all, this is a study carried out with subjects with no history of violence; intimate partner batterers have a distinct psychological profile (Shorey et al., 2012a), and multiple factors play a role in their perception of violence (Riggs and O’Leary, 1989; Bell and Naugle, 2008), power (Straus, 1976; Sagrestano et al., 1999) and their often repudiated need of help, denial and minimization (Henning et al., 2005). Overall, these issues can bring about unexpected results when paradigms tested in volunteers who have not had experience of violent situations extend to the clinical and forensic realms. However, encouraging results have already been obtained (Seinfeld et al., 2018), suggesting that even though forensic populations have different psychological profiles, they may still profit from setups such as that described here. Another issue to take into consideration is that participants underwent either one condition or the other, but they did not experience both perspectives so that the subjective appraisal and the behavioral and physiological reactions to one condition are not relative to the other. This was an essential part of the experimental design though, to avoid habituation effects and demand characteristics, where subjects would easily understand the point of the experiment. Also, 1PP and 3PP are not symmetrical experiences—the experience of one is likely to influence the experience of the other—and no amount of randomization of order can overcome this point (i.e., there would be two distinct groups depending on order).

To conclude, we have shown that an immersive virtual reality scene of intimate partner violence of men toward women could be used in the rehabilitation programs of batterers, especially if it is experienced from the 1PP of the female victim. Our paradigm is based on the impact that virtual embodiment and perspective changing has on our experiences and subsequent behavior. In this case, the scene was designed for male abusers and female victims but could be eventually adapted to other forms of intimate partner violence (female abusers or same-sex violence) and other forms of domestic violence (e.g., violence to children) and, more generally, for violent behavior. We propose that the efficacy of immersive virtual worlds for clinical purposes arises from the contribution of multiple factors that induce the feeling of realness, presence and identification with the virtual characters, all of which tend to be higher if experienced from the 1PP of the character of interest, in a design that incorporates visuomotor and visuotactile synchrony between the real and the virtual bodies. Finally, given the worldwide problem of intimate partner violence represents (World Health Organization [WHO], 2013), it is of utmost importance to develop resources for victims, but also for perpetrators with the aim of reducing domestic violence by making them more empathic, by changing implicit attitudes and by training for non-violent responses during intimate partner interactions.

## Data Availability Statement

The datasets generated for this study are available on request to the corresponding author.

## Ethics Statement

The studies involving human participants were reviewed and approved by the Ethics Committee of the Hospital Clínic de Barcelona. The patients/participants provided their written informed consent to participate in this study.

## Author Contributions

LZ, MS, and MS-V designed the experiments. GI and DB generated the virtual environments. LZ, GI, SS, and J-AP ran the experiments. LP-M and JA-P analyzed the physiological data. CG-L and LZ analyzed the other measures. CG-L wrote the first draft. All authors contributed to the final version of the manuscript. MS-V supervised the research.

## Conflict of Interest

MS-V and MS are founders of Virtual Bodyworks Inc. This work is not funded nor influenced in any form by Virtual Bodyworks. The remaining authors declare that the research was conducted in the absence of any commercial or financial relationships that could be construed as a potential conflict of interest.
